# Trans-Biliary Gastric Outlet Recanalization and Stenting: A Case Report

**DOI:** 10.7759/cureus.22692

**Published:** 2022-02-28

**Authors:** Shahbaz Qazi, Mohamed R Elzahrani, Abdullah T Tatwani, Ahmed S Hilabi

**Affiliations:** 1 Radiology, King Abdulaziz Medical City, Riyadh, SAU; 2 Radiology, College of Medicine - King Saud Bin Abdulaziz University for Health Sciences, Riyadh, SAU; 3 Medical Education, College of Medicine - King Saud Bin Abdulaziz University for Health Sciences, Riyadh, SAU; 4 Medicine, College of Medicine - King Saud Bin Abdulaziz University for Health Sciences, Riyadh, SAU

**Keywords:** multiple co-morbidities, interventional radiology, malignant gastric outlet obstruction, recanalization, trans-biliary

## Abstract

Gastric ischemia is a condition of hypo-perfusion associated with hypotension, vasculitis, and thromboembolism. We report a case of a gastric outflow obstruction due to sizeable visceral artery thrombo-embolism leading to the ischemic conclusion, the frailty, multiple comorbidities deeming general anesthesia (GA) risky, and the patient’s decision not to have an open surgery under GA. Invasive procedures in patients with similar profiles like our patient are usually not risk-free, this leads the intervention radiology team to believe a minimally invasive procedure while avoiding GA might be optimal. A 63-year-old female with multiple comorbidities came eight weeks after significant surgery complaining of severe acute epigastric pain, abdominal distention and rigidity, and persistent vomiting. Further investigations showed obstruction in the gastric antrum and pyloric canal. Three separate endoscopic attempts to find and cross the stricture failed. Firstly, gastrostomy access was established, but due to the stomach being massively distended, passing a guidewire through the pylorus failed despite using multiple hydrophilic wires and pre-shaped catheters, this is due to the collapsed pylorus. Subsequently, two attempts under ultrasound guidance to puncture the duodenal bulb and pass a wire and catheter through the antrum stricture were unsuccessful, and another attempt was considered of high risk. An alternative approach through the gallbladder was established, and cholangiography was performed to delineate the anatomy. Then an approach through the right hepatic duct and ampulla of Vater was successfully performed. The attempted passage through the stricture was successful. The dilation was successful, and the patient tolerated both fluid and solids orally. Due to having such a frail patient, interventions of minimal invasiveness and favorable outcome are welcomed. This case report suggests that this technique showed satisfactory results and achieved the goal to improve the overall quality of life where the patient had a good oral intake with no post-operation complications.

## Introduction

Gastric ischemia is a product of generalized or localized vascular insufficiency caused by various etiologies, such as systemic hypotension, vasculitis, disseminated thromboembolism, or mesenteric stenosis [1]. It can cause significant morbidity through persistent nausea and vomiting and debilitating effect on the quality of life [2]. Gastric outlet obstructions are a common result of gastric ischemia [3]. The most common etiology of gastric ischemia used to be peptic ulcer diseases (PUD) [4], but in recent decades malignant causes account for 50-80% of cases [5]. Almost half of the overall patients involved in such cases require non-conservative management [6]. This might include intervention and surgical techniques. Survival rates of gastric ischemia are not the concern in most patients [7], overall quality of life is [3]. In this case report, the authors report chronic gastric outlet ischemic stricture secondary to large visceral arteries thromboembolism and subsequent recanalization via trans-biliary approach after failed endoscopic and trans-gastric attempts [8]. Thus, the retrograde trans-biliary recanalization technique allows a safe gastric stent placement with the assisted trans-gastric approach. Technical success rates of endoscopically inserted stents are the same as those of radiologically inserted stents. If endoscopic approaches fail, radiographic approaches might be attempted and vice versa [8].

## Case presentation

A 63-year-old woman is a known case of diabetes mellitus, hypertension, achalasia, depression, dyslipidemia, hypothyroidism, and a previous cerebrovascular accident (CVA). Past surgical history is significant for a history of ileal loop ischemia with bowel perforation secondary to celiac artery, superior mesenteric, and inferior mesenteric artery thrombosis two months prior. The event was managed by emergent small bowel resection with repair and end ileostomy creation combined with surgical thrombo-embolectomy of the superior mesenteric artery. After the surgery, by eight weeks, she presented to the emergency room with severe acute epigastric pain with abdominal distension, diffuse pain in the abdomen, abdominal rigidity, and persistent vomiting, and the patient was vitally stable with a negative systemic review. Initial investigations included complete blood count (CBC), venous blood gas (VBG), urine analysis, troponin I, creatinine kinase, blood type compatibility, chest X-ray, abdomen, and pelvis computerized tomography (CT). Upper gastrointestinal study and images, CT and fluoroscopy, showed severe narrowing of the second part of the pyloric canal with luminal occlusive stricture at the gastric antrum with massive gastric dilatation. In the meantime, the patient received nourishment through a central total parenteral nutrition (TPN) via a peripherally inserted central venous catheter (PICC) line. Endoscopic evaluation failed to find and cross the gastric stricture through three separate attempts. Due to the patient’s frail condition from the recent major surgery and the patient’s refusal to undergo open surgery, the patient consented to let the interventional radiology team interfere.

Intervention

**Figure 1:**
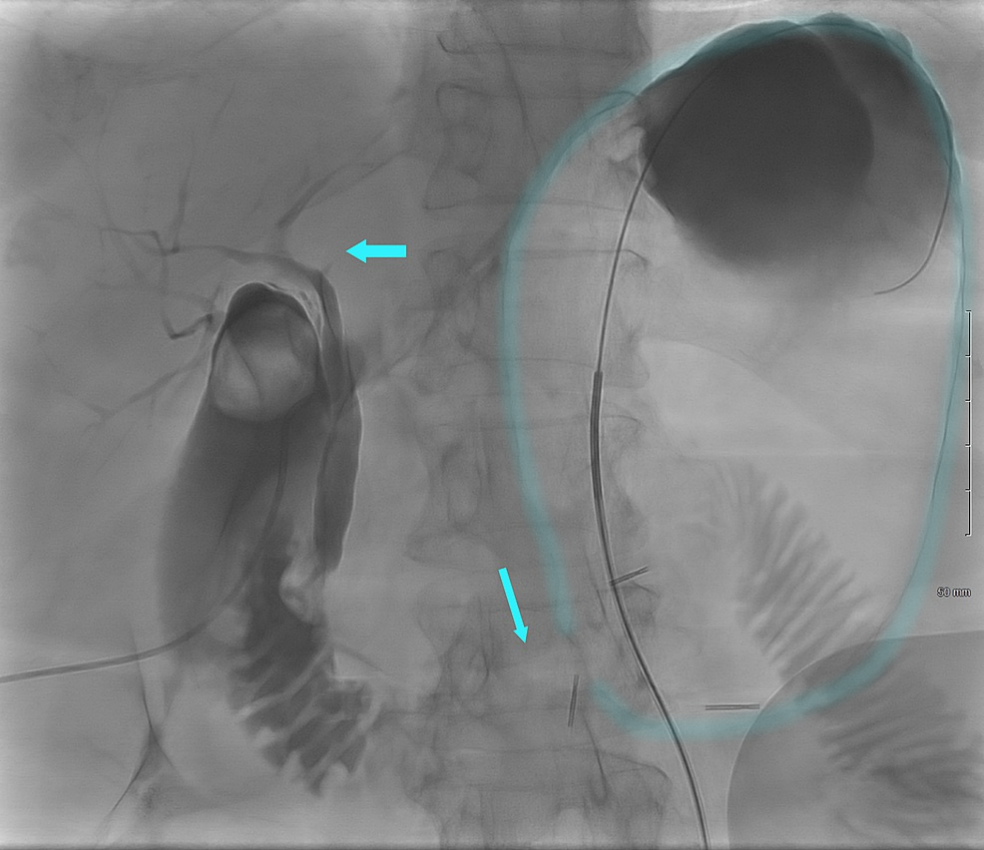
Cholecysto-cholangiogram demonstrates an intrahepatic channel and distended obstructed stomach. Gastric stenosis is demonstrated in this image. Imaging modality: Fluoroscopy showing a distended stomach with an accumulation of contrast material.

**Figure 2:**
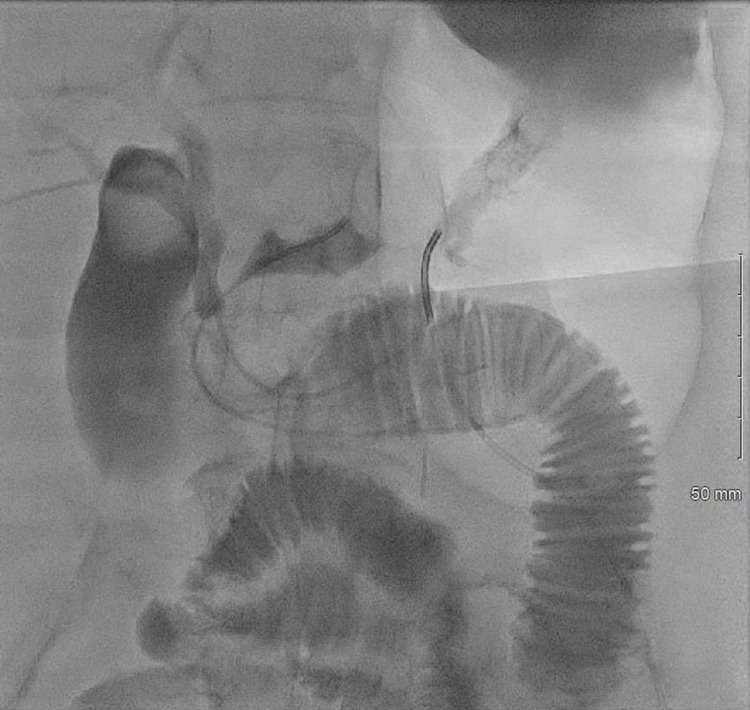
Via transhepatic approach catheter has been placed through the trans-biliary channels across the pylorus of the stomach. Imaging modality: Fluoroscopy.

In our patient, the original obstruction was at the junction of the gastric outlet extending along the first part of the duodenum. Given this, the stent was placed initially across this point, extending into the second part of the duodenum. The gastric outlet obstruction at the top end required the second stent to be placed with the proximal end in the gastric pylorus. A transhepatic stent was not considered because it required a 10-French (F) tract through the liver [4]. Transoral stent placement enabled the transhepatic tract to be maintained on 4-F. The stent was introduced through the oropharynx, esophagus, and stomach, allowing the passage of a 10-F sheath without dilation of a non-physiologic tract [4]. At first, gastrostomy access was established after placing three gastric anchor sutures under general anesthesia. The access was proven difficult due to many failed trials of passing through the gastric outlet obstruction despite using different hydrophilic wires and pre-shaped catheters, where the difficulty that has been encountered was within the stomach being massively distended, making the manipulation of the guidewire through the pylorus tedious and time-consuming (Figures 1, 2) [5].

**Figure 3:**
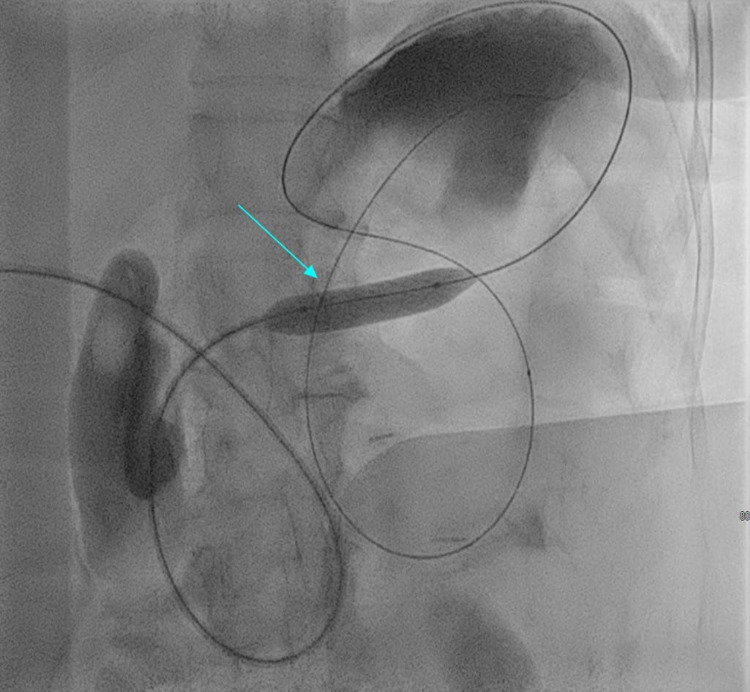
Pyloric stricture that has been crossed by the wire, and the wire was placed in the stomach and was dilated via a balloon. Imaging modality: Fluoroscopy. The arrow indicates the balloon used to dilate the stricture prior to stent placement.

Due to the failure to pass the gastric outlet, two attempts were made under ultrasound guidance to puncture the duodenal bulb to steer the wire and catheter to cross the gastric antrum stricture. Both attempts failed because of peristalsis. The duodenal approach was deemed risky and could not tolerate any other attempts. Alternatively, an ultrasound-guided puncture of the gallbladder with a 21-gauge needle with the placement of Three French inner sheath dilators over the 0.18 wire was performed. Cholangiography was performed to outline the intrahepatic duct channels. Then using a transhepatic approach with a 21-gauge needle using an acoustic micro-puncture kit (Boston Scientific, Marlborough, MA, USA), access to the right hepatic duct was successfully made. Using a 4-F Cobra 1 catheter (Cordis, Johnson & Johnson, Brentford, United Kingdom), an angled 0.035-inch 145-cm guidewire (Terumo, Tokyo, Japan) was manipulated through the ampulla of Vater and then retrogradely attempted to cross the stricture with a wire. A second attempt with a six French Terumo destination straight tip 45 cm sheath was placed successfully close to the duodenal bulb. Using a 0.35 stiff glide wire with J shaped tip 260 cm long with a 100-cm 5 French Kumpe catheter, we could navigate and cross the tight stricture then push the wire deep into the stomach, making a loop as seen in Figure 3.

**Figure 4:**
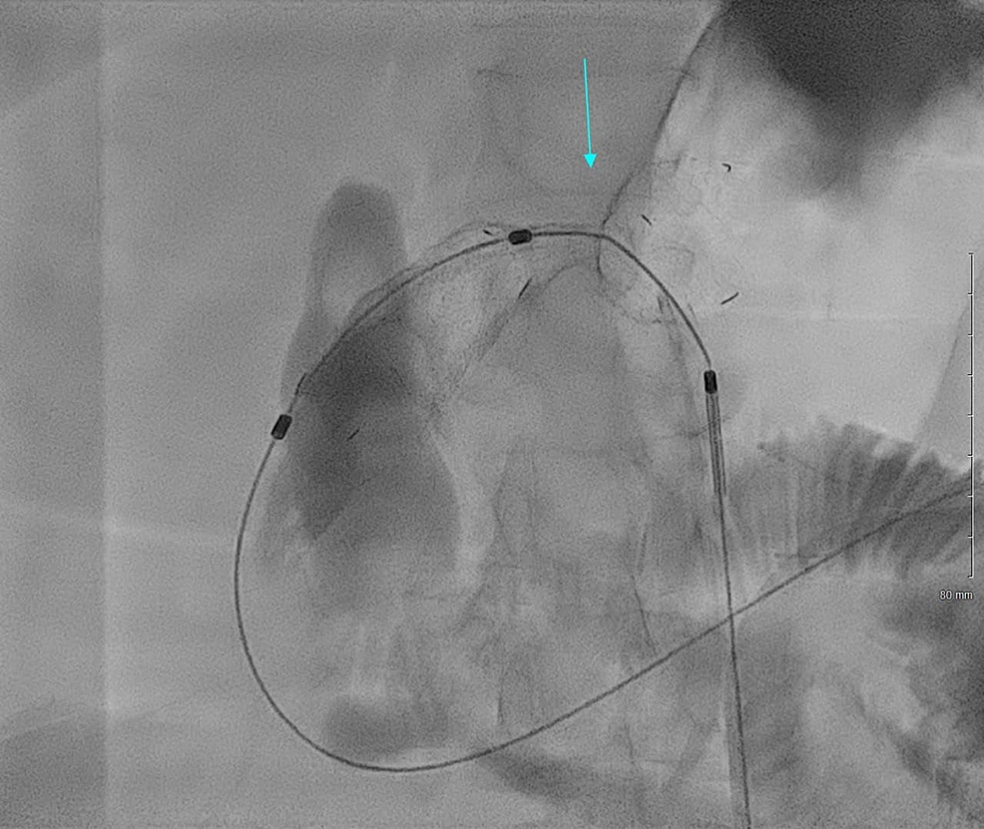
Using an abdominal trans-gastric wall approach a stent was placed, which showed a very tight waist. Imaging modality: Fluoroscopy. The arrow indicates the narrowest part of the gastric outlet after the initial stent placement.

A balloon dilatation with a size of 12mm x 4cm (Mustang, Boston Scientific) was placed to secure the gastric stricture track. The wire and balloon catheter were approximated to the fundus of the stomach to secure access. Then, an Amplatz Goose Neck snare (EV3, Plymouth, MN, USA) was used via a trans-gastric approach. Afterward, an endoscopic six French alligator forceps was used successfully to catch the catheter and withdraw it from the stomach through the gastrostomy access. Through-and-through 260-cm stiff Amplatz wire (EV3, Plymouth, MN, USA) was passed and secured from both ends (Figure 4).

**Figure 5:**
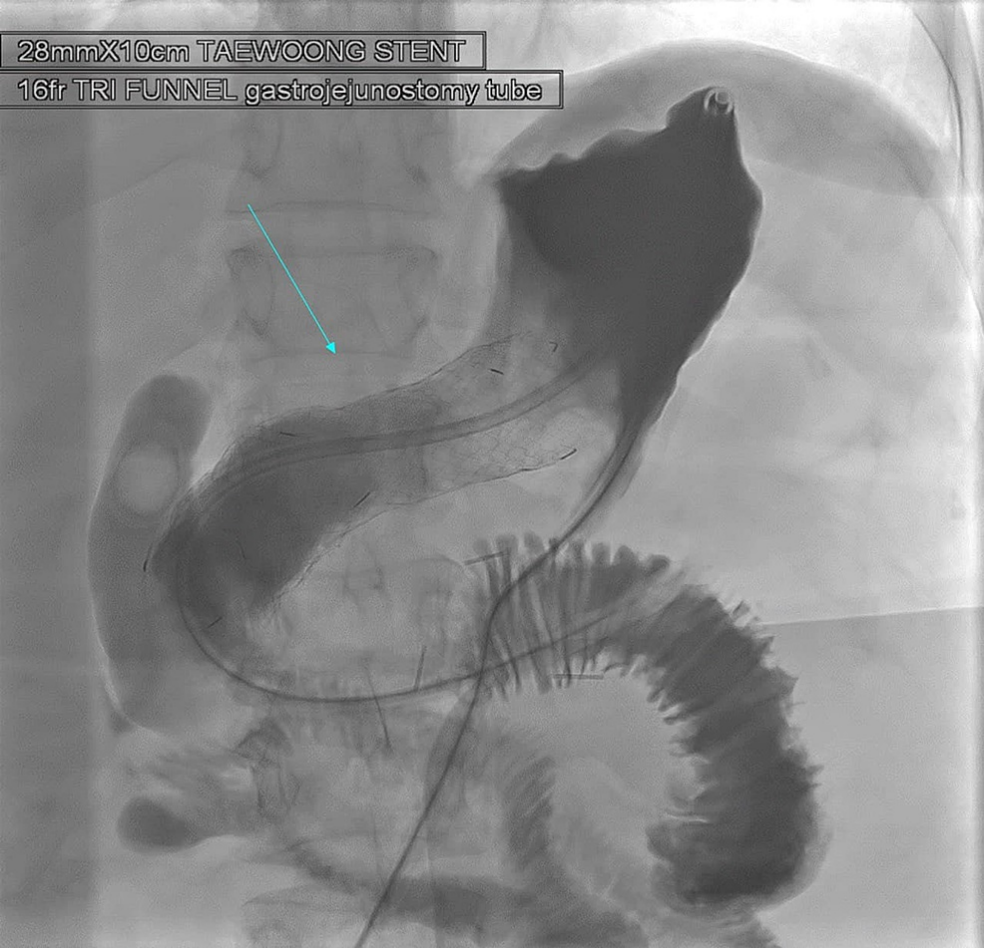
Using a trans-gastric approach, a 14 French gastro-jejunostomy catheter was placed with a top in the jejunum. Imaging modality: Fluoroscopy. The arrow is approximately in the same area as in Figure 4, now visibly patent after successful dilation.

Furthermore, a 0.018-inch transcend wire was pushed in a 5F Bern catheter antegrade towards the jejunal loops for security. Eventually, the trans-biliary access was abandoned, and 260-cm Amplatz wire was secured deep in the jejunal bowel loop. Finally, a 28mm x 10cm uncovered stent Niti-S™ D Pyloric/duodenal Stent (Taewoong, South Korea) was then satisfactorily positioned across the stricture. There was a very tight waist which was dilated with a 24mm x 4cm balloon. Through gastrostomy access, 16 French tri funnel gastrojejunostomy catheter was placed with a tip in the jejunal loop, and the biliary access was no longer required to remove it. Post recovery the patient was tolerating fluids and solids, and she suffered no complications following the procedure (Figure 5).

**Figure 6:**
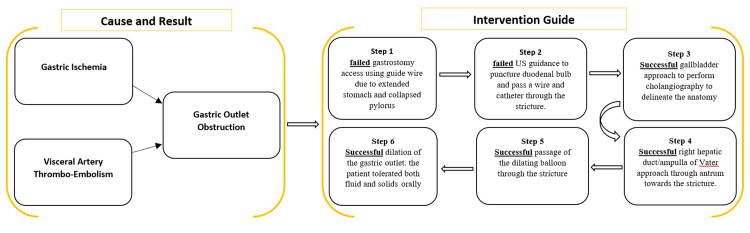
Schematic summary showing the etiology of gastric outlet obstruction and interventional steps. Summary for better visualization and understanding of the therapeutic phase.

To better understand the overall therapeutic phase/steps, please check the scheme in Figure 6.

## Discussion

In gastric stricture, the usual feeding route is a nasogastric tube or TPN. TPN is used as the nasogastric tube upsets the quality of life for many patients. Furthermore, the collateral blood supply to the stomach is protective from thromboembolic events [9]. However, in events involving all major vessels supplying the bowel, as in the case of our patient, gastric ischemia occurs. Non-surgical options include endoscopic or radiologic placement of a duodenal stent. It is usually feasible to cross most strictures with a guidewire by using a coalescence of fluoroscopy and endoscopy. This, however, may not be possible with very tight stenoses, in which case a surgical bypass may be needed [10]. Although endoscopically placed stents should be the ideal standard of care by a gastroenterologist, where endoscopy can be used for bowel decompression, dilation of strictures, or placement of self-expandable metal stents to restore the luminal flow either as a definitive treatment or to allow for a delay until elective surgical therapy [11]. Failure of such a procedure leads an interventional radiologist to intervene. Many options exist for the interventional radiology team to choose from, such as transhepatic duodenal stent placement which has previously been described with oral assistance. Our technique describes a combined transhepatic and trans-gastric approach for placing a gastric stent as a novel solution. The stent placement is an alternative to surgery in patients with a high comorbidity burden with good clinical outcomes [12, 13].

Moreover, clinical awareness of this spectrum of situations will allow interventional radiologists to appropriately diagnose, plan, and manage the affected patient in a proper way. Wide-bore stents had always been a better profile in preventing restenosis and relieving occlusive symptoms. Also, it helps in the early emptying of food materials and improves the quality of life (QOL). Due to comorbidities, refusal of open surgery, and failed endoscopic trans-gastric attempts, this interventional technique was offered. Self-expandable metallic stents are effective in palliation, offering a rapid relief of benign gastric outlet obstruction [10, 12, 14]. Thus, they should be contemplated in patients with poor prognosis, whereas patients with a fairer prognosis should be offered a surgical bypass [15]. The radiographic stenting approach is not without any cons. These include the high cost of therapy, which cannot be tailored to all patients, often neglected as an initial option due to surgeons not informing the patients about other emerging novel approaches. On the other hand, the pros of using this approach include a high success rate, low mortality, and good clinical outcome in terms of improving the QOL of the patient [16].

Likewise, in our case, that is presented the patient had multiple comorbidities where open surgery was not applicable due to the general anesthesia that will increase the risk of mortality. Thus, the case was inevitably given to the interventional radiology department to perform the procedure. The limitations of the study included the lack of generalization of the case, there was only one patient included in the study, and the lack of long-term follow-up for this patient.

## Conclusions

Our case report is limited to just one patient. It would be beneficial to evaluate results over a more extensive series, but this would be difficult because patients with ischemic gastric outflow obstruction have a short life expectancy due to advanced disease and are usually treated conservatively after restenosis of the stent to their frail condition. However, for our patient, this technique showed satisfactory results, and it achieved the goal to improve the patient’s overall quality of life where the patient had good oral intake and suffered no adverse effects post-operation.

## References

[1] Saldaña Dueñas C, Elosua González A, Guerra Lacunza A (2018). Gastric ischemia due to critical stenosis of the celiac trunk. An Sist Sanit Navar.

[2] Mehta S, Hindmarsh A, Cheong E (2006). Prospective randomized trial of laparoscopic gastrojejunostomy versus duodenal stenting for malignant gastric outflow obstruction. Surg Endosc.

[3] Tringali A, Giannetti A, Adler DG (2019). Endoscopic management of gastric outlet obstruction disease. Ann Gastroenterol.

[4] Cherian PT, Cherian S, Singh P (2007). Long-term follow-up of patients with gastric outlet obstruction related to peptic ulcer disease treated with endoscopic balloon dilatation and drug therapy. Gastrointest Endosc.

[5] Johnson CD (1995). Gastric outlet obstruction malignant until proved otherwise. Am J Gastroenterol.

[6] Sharma A, Mukewar S, Chari ST, Wong Kee Song LM (2017). Clinical features and outcomes of gastric ischemia. Dig Dis Sci.

[7] Jaffin BW, Kaye MD (1985). The prognosis of gastric outlet obstruction. Ann Surg.

[8] Lopera JE, Brazzini A, Gonzales A, Castaneda-Zuniga WR (2004). Gastroduodenal stent placement: current status. Radiographics.

[9] Tang SJ, Daram SR, Wu R, Bhaijee F (2014). Pathogenesis, diagnosis, and management of gastric ischemia. Clin Gastroenterol Hepatol.

[10] Milson A, Abdellaoui A, Fraser C, Watkinson AF (2010). Transhepatic assisted transoral placement of a duodenal stent in malignant gastric outlet obstruction. J Vasc Interv Radiol.

[11] Aviv RI, Shyamalan G, Khan FH, Watkinson AF, Tibballs J, Caplin M, Winslett M (2002). Use of stents in the palliative treatment of malignant gastric outlet and duodenal obstruction. Clin Radiol.

[12] Gowen GF (1985). Endoscopic decompression in partial small bowel obstruction. Am J Surg.

[13] AlGharras A, Dey C, Molla N (2021). Transhepatic approach for retrograde D2 duodenal stent placement: new technique and case series. J Vasc Interv Radiol.

[14] Salminen P, Gullichsen R, Laine S (2009). Use of self-expandable metal stents for the treatment of esophageal perforations and anastomotic leaks. Surg Endosc.

[15] Hucl T (2013). Acute GI obstruction. Best Pract Res Clin Gastroenterol.

[16] Vlavianos P, Zabron A (2012). Clinical outcomes, quality of life, advantages and disadvantages of metal stent placement in the upper gastrointestinal tract. Curr Opin Support Palliat Care.

